# Profiling novel pharmacology of receptor complexes using Receptor-HIT

**DOI:** 10.1042/BST20201110

**Published:** 2021-08-26

**Authors:** Elizabeth K.M. Johnstone, Kevin D.G. Pfleger

**Affiliations:** 1 Molecular Endocrinology and Pharmacology, Harry Perkins Institute of Medical Research, QEII Medical Centre, Nedlands, Western Australia 6009, Australia; 2 Centre for Medical Research, The University of Western Australia, Crawley, Western Australia 6009, Australia; 3 Australian Research Council Centre for Personalised Therapeutics Technologies, Australia; 4 Dimerix Limited, Nedlands, Western Australia 6009, Australia

**Keywords:** BRET, G-protein-coupled receptors, intracellular signaling, receptors

## Abstract

Many receptors are able to undergo heteromerisation, leading to the formation of receptor complexes that may have pharmacological profiles distinct from those of the individual receptors. As a consequence of this, receptor heteromers can be classed as new drug targets, with the potential for achieving greater specificity and selectivity over targeting their constituent receptors. We have developed the Receptor-Heteromer Investigation Technology (Receptor-HIT), which enables the detection of receptor heteromers using a proximity-based reporter system such as bioluminescence resonance energy transfer (BRET). Receptor-HIT detects heteromers in live cells and in real time, by utilising ligand-induced signals that arise from altered interactions with specific biomolecules, such as ligands or proteins. Furthermore, monitoring the interaction between the receptors and the specific biomolecules generates functional information about the heteromer that can be pharmacologically quantified. This review will discuss various applications of Receptor-HIT, including its use with different classes of receptors (e.g. G protein-coupled receptors (GPCRs), receptor tyrosine kinases (RTKs) and others), its use to monitor receptor interactions both intracellularly and extracellularly, and also its use with genome-edited endogenous proteins.

## Introduction

Receptors are proteins that enable transduction of signals from the extracellular environment, as well as between and within cells [1], and they play crucial roles in cellular and organismal physiology. Many receptors are transmembrane proteins, such as G protein-coupled receptors (GPCRs) and receptor tyrosine kinases (RTKs), while others are soluble proteins, such as nuclear receptors and some cytokine receptors [2]. Oligomerisation is a feature of many receptors, and for some, it is obligate, meaning that it is required for the formation of a fully functional receptor. These types of receptor oligomers have been classified as ‘homomeric receptors’ or ‘heteromeric receptors’ [3], and RTKs are the archetypal example of these, requiring homo- or hetero-merisation for initiation of signalling [4]. Another classic example of an obligate receptor oligomer is the heteromeric γ-aminobutyric acid (GABA) GPCR, which only forms a functional receptor upon heteromerisation of both GABA_B1_ and GABA_B2_ receptor units [5–7]. In contrast, there are also ‘receptor homomers’ and ‘receptor heteromers’, which are composed of functional receptors and display novel pharmacology (known as their biochemical fingerprint) that is distinct from their constituent receptors [3]. Non-obligate receptor oligomers, and in particular receptor heteromers, have been extensively studied, particularly in relation to GPCRs, and there are many examples of GPCR heteromers reported in the literature [8,9]. These non-obligate receptor oligomers are particularly interesting entities, as their attainment of novel pharmacology substantially increases the complexity of their receptor signalling systems. Furthermore, they can be considered novel drug targets, with the potential to obtain improved specificity and selectivity over targeting the individual receptors. However, receptor homomers and receptor heteromers have been largely overlooked by the pharmaceutical industry. One reason for the lack of research into non-obligate oligomers, and specifically heteromers, is that they are relatively difficult to investigate. This is because differentiating heteromer-specific pharmacology from monomer- or homomer-specific pharmacology can be a challenge. To address this, our laboratory developed the Receptor-Heteromer Investigation Technology (Receptor-HIT) [10,11].

**Figure 1.**
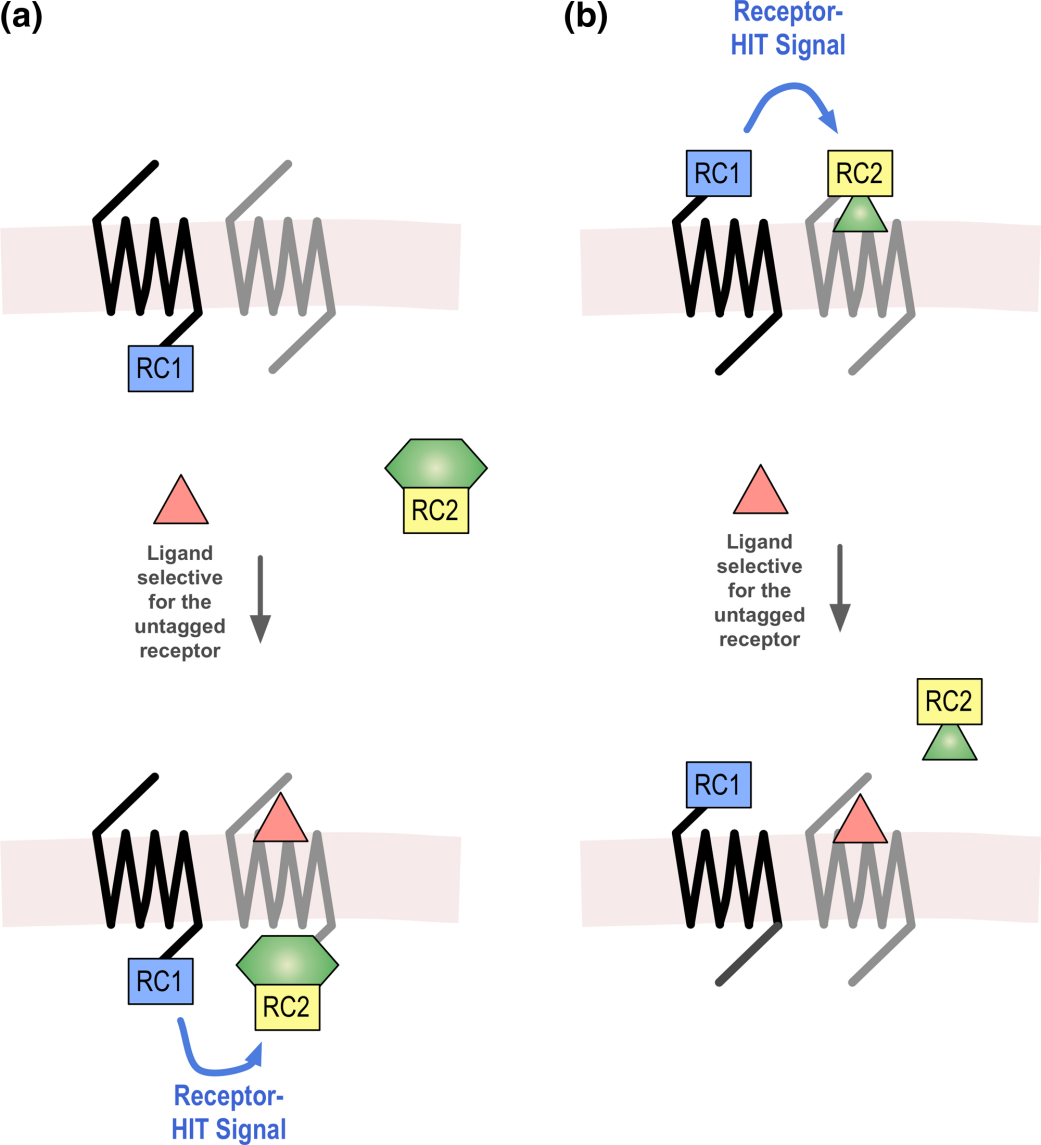
Intracellular and extracellular Receptor-HIT assay. The Receptor-HIT assay uses a proximity-based reporter system such as bioluminescence resonance energy transfer (BRET) to monitor ligand-dependent modulation of interactions between receptors and specific biomolecules. The assay works by coexpressing both receptors, one unlabelled and the other labelled with the first reporter component of the proximity assay (RC1), such as a BRET donor. The second reporter component (RC2), such as a BRET acceptor, is used to label an interacting biomolecule. Usually, this interacting biomolecule is a coexpressed intracellular protein (**a**) however it can also be a labelled ligand (**b**). The assay works by adding a ligand that is selective for the untagged receptor. If this results in modulation of the proximity signal between the tag on the receptor and the tag on the interacting biomolecule, this indicates heteromerisation of the two receptors. Figure reproduced from [53].

Receptor-HIT is an assay that allows detection and characterisation of heteromers through ligand-induced recruitment of biomolecules, utilising a proximity-based assay as a reporter system. The Receptor-HIT assay comprises four components: a labelled receptor, an unlabelled receptor, a labelled interacting biomolecule, and a ligand that is specific for the unlabelled receptor (or the heteromer; Figure 1). The assay works by coexpressing both receptors in cells, and monitoring the proximity of the labelled interacting biomolecule with the labelled receptor upon addition of a ligand that is selective for the unlabelled receptor. A Receptor-HIT signal is generated when the proximity signal between the labelled interacting biomolecule and the labelled receptor has been modulated by treatment with the ligand for the unlabelled receptor (or the heteromer). This Receptor-HIT signal is indicative of heteromerisation as the most likely explanation for a proximity signal being generated is that the interacting biomolecule is brought into close proximity to the labelled receptor because it interacts with the activated unlabelled receptor that is itself in close proximity to the labelled receptor. It is also possible that activation of the unlabelled receptor results in transactivation of the labelled receptor, with consequent recruitment of the interacting biomolecule to the labelled receptor. A good example of when this is believed to occur is with the α_1A_-adrenoceptor-CXC chemokine receptor 2 heteromer [12]. This is still likely to be due to the receptors being present together in a complex, which can be substantiated by other approaches that report on receptor proximity rather than functional interaction [12]. However, direct physical contact between the receptors is not required and may be mediated by other molecules that enable the functional interaction. Most commonly, Receptor-HIT has been used to investigate interactions that occur at the intracellular regions of receptors, using signalling or regulatory proteins as the interacting biomolecules (Figure 1a). However, it can also be used to investigate interactions that occur extracellularly, using labelled ligands as the interacting biomolecules (Figure 1b).

Receptor-HIT can be used on various proximity-based platforms, but has most commonly been used with bioluminescence resonance energy transfer (BRET). Other suitable platforms include fluorescence resonance energy transfer, protein complementation (such as bimolecular fluorescence or luminescence complementation or enzyme fragment complementation) and the protease cleaved transcription factor assay system known as Tango^TM^ [13,14].

Receptor-HIT's major strength is that it enables the characterisation of heteromer-specific pharmacology, its biochemical fingerprint, without requiring the functioning of either receptor to be impaired. Furthermore, particularly when using BRET, it allows characterisation in real-time and in live cells. Identification and characterisation of this biochemical fingerprint in recombinant systems can be the first step in the process of confirming the existence of the heteromer in native systems. By establishing the heteromer-specific pharmacology, this can be used in native systems to differentiate heteromer-induced responses from responses induced by the constituent receptors, thereby providing evidence of the heteromer's existence in native systems. It is important to appreciate that in addition to Receptor-HIT, there are multiple other techniques used for detecting and characterising receptor complexes, which include *in situ* hybridisation, immunohistochemistry, proximity ligation assays and coimmunoprecipitation [8]. The use of a variety of approaches gives additional support to the existence of a receptor complex. However, it is also important to note that the proximity of receptors, or even their presence in the same macromolecular complex, does not necessarily mean they are functionally interacting to influence each other's pharmacology. The heteromer assay developed by van Rijn et al. [15], based on the homomer assay published by Han et al. [16], provides evidence of functional interaction. However, it requires the use of highly engineered truncated receptor-G protein fusions that render the receptor unable to signal in its own right, such that the G protein requires the presence and activation of another receptor to generate a signal. This is an interesting addition to the toolbox for studying heteromers, although the impact of ‘muting' one of the receptors on the physiological functioning of the heteromer is unclear.

As Receptor-HIT is used in recombinant systems, it has most commonly been used with overexpressed proteins. However, it is sensitive enough to be used with proteins at very low expression levels, and has been demonstrated with proteins expressed at endogenous levels [17] (discussed in more detail later). When conducting Receptor-HIT assays, the ratio of the two receptors of interest, as well as the ratio of receptors and labelled interacting partner, need to be considered. Often, the ratio of choice is empirically determined through optimisation studies, and the physiological relevance of this needs to be considered when extrapolating results into native systems.

This review will discuss a selection of the studies that have utilised the Receptor-HIT assay on the BRET platform. These studies include heteromers composed of several different classes of receptors that have been investigated with various adaptations of the Receptor-HIT assay.

## GPCR-GPCR Receptor-HIT

Receptor-HIT has most commonly been used to investigate the pharmacology of receptor heteromers composed of two different GPCRs (termed GPCR-HIT) [10,12,17–24]. GPCRs are the largest family of membrane receptors and are also the largest family of drug targets, with over 30% of currently approved drugs acting via GPCRs [25–27]. Traditionally, Family A GPCRs in particular were believed to only function as individual monomeric units. However, over the past several decades, mounting evidence has revealed that even these GPCRs are able to also function as higher-order oligomers, including homomers and heteromers [9].

Currently, the most clinically advanced output of a study that has used Receptor-HIT is the heteromer between the angiotensin II (AngII) type 1 (AT_1_) receptor and the chemokine CCR2 receptor. Studies have shown that interactions between the signalling pathways of the endogenous ligands for each receptor (AngII and CCL2, respectively) are involved in the inflammation and vascular remodelling associated with hypertension [28,29] and hypertensive nephropathy [30], as well as atherosclerosis and abdominal aortic aneurysms [31,32]. Furthermore, functional interactions between the receptors themselves have also been reported, with studies showing that dual receptor antagonism significantly improved outcomes of ischemic brain damage [33] and renal injury in antiglomerular basement membrane nephritis [34]. These studies supported our hypothesis that these receptors functionally interact, as we demonstrated using Receptor-HIT in HEK293FT cells [21]. The Receptor-HIT assay indicated that AngII-induced activation of the unlabelled AT_1_ receptor, which primarily couples to Gα_q_, resulted in inhibition of CCR2-Gα_i_ coupling. We also observed a Receptor-HIT signal in the form of AngII-induced recruitment of β-arrestin2/Venus to CCR2/Rluc8, only when it was coexpressed with the unlabelled AT_1_ receptor. Furthermore, treatment with agonists for both receptors resulted in a BRET signal from β-arrestin2/Venus recruitment to CCR2/Rluc8 that was more than additive of the signals resulting from each agonist alone, and dual receptor antagonism was required to completely abolish this signal. As we observed this synergistic inhibition upon dual receptor antagonism, we then followed up these *in vitro* results by comparing the effects of dual receptor blockade to individual receptor blockade in a subtotal-nephrectomised (STNx) rat [21]. The STNx model involves right subcapsular nephrectomy (removal of the right kidney) and infarction of ∼2/3 of the left kidney (where necrosis of the tissue due to lack of oxygen is caused by cutting off the blood supply to 2/3 of the left kidney) [35]. This leaves 1/6 kidney function and is a model that closely resembles human chronic kidney disease. We found that dual receptor blockade resulted in significant decreases in proteinuria, reductions in podocyte loss and prevention of renal injury independent of blood pressure, compared with individual antagonists alone. As a result of these findings, a Phase 2a clinical trial in patients with a range of chronic kidney diseases was undertaken. Subgroup analysis of this study (completed in 2017) revealed that dual receptor antagonism with AT_1_ inhibitor irbesartan and CCR2 inhibitor DMX-200 resulted in statistically and clinically significant efficacy signals in diabetic patients, and led to two more Phase 2 studies, in patients with diabetic kidney disease and Focal Segmental Glomerulosclerosis (FSGS). Completed in July 2020, the FSGS trial results showed that 86% of the patients demonstrated a reduction in proteinuria (protein in the urine) when DMX-200 was given in addition to irbesartan, versus placebo (stable irbesartan treatment). Completed in September 2020, the Phase 2 diabetic kidney disease trial sub-group analysis showed statistically and clinically significant improvement for patients with higher levels of albuminuria (albumin in the urine) at study baseline (>500 mg/g). Furthermore, in 2020, DMX-200 was selected for global studies to treat Acute Respiratory Distress Syndrome (ARDS) in COVID-19 patients (REMAP-CAP) and COVID-19-induced respiratory complications (CLARITY 2.0) due to the involvement of both AT_1_ and CCR2-mediated pathways in lung inflammation associated with infection with the SARS-CoV2 virus. The progression of this story from the initial *in vitro* Receptor-HIT studies to Phase 3 clinical trials highlights the value of the Receptor-HIT assay for early-stage drug discovery for detection and characterisation of heteromers.

## GPCR-other receptor Receptor-HIT

Receptor-HIT has also been used to investigate heteromers composed of GPCRs and other receptors, including RTKs such as epidermal growth factor receptor (EGFR) [36,37], vascular endothelial growth factor receptor 2 (VEGFR2) [38], and the insulin receptor [39], as well as the receptor for advanced glycation end-products (RAGE) [40].

The formation of heteromeric complexes between RTKs and GPCRs has been well described in the literature and is commonly known as transactivation [41]. A good example is the transactivation of the EGFR by the AT_1_ receptor, which mediates cell growth and survival, and has been implicated in numerous cardiovascular pathologies [42]. Using the Receptor-HIT assay in various immortalised and primary cells, we demonstrated AngII-induced recruitment of Grb2/Venus proximal to EGFR/Rluc8 and showed that the transactivation was independent of G_q/11_ and β-arrestin, and only partially dependent on EGFR tyrosine kinase activity [36]. We then followed up this study by investigating the involvement of a protein called TRIO in AT_1_-mediated EGFR transactivation, as it had previously been identified as an intermediate specific to AngII/AT_1_-mediated EGFR transactivation rather than EGF-mediated EGFR transactivation [43]. Using the Receptor-HIT assay to investigate the role of TRIO in AT_1_-EGFR transactivation, we found that Rluc8/TRIO was recruited to EGFR/Venus only upon coexpression and activation of the AT_1_ receptor, and that this was also dependent on G_q/11_ activity [37]. We also demonstrated other AngII/AT_1_- and G_q/11_-dependent aspects of TRIO pharmacology, including cellular trafficking and interactions with other signalling proteins including GRK2, PKCδ and Gγ_2_.

RAGE is a transmembrane receptor of the immunoglobulin super family, and is a key regulator of the innate immune response [44]. RAGE signalling is up-regulated following inflammation, and it is associated with numerous diseases, including diabetes, cardiovascular disease, cancer and Alzheimer's disease [44]. Typically, RAGE inflammatory signalling has been ascribed to activation of the RAGE extracellular domain by ligands such as S100 proteins (also known as calgranulins) and advanced glycation end-products (AGEs). However, we found that the cytoplasmic domain of RAGE can also be transactivated by the AT_1_ receptor, in a cognate-ligand independent manner, and that rather than the classical G_q_ pathway, this transactivation pathway mediates much of the inflammation caused by AT_1_ receptor activation [40]. In various *ex vivo* and *in vitro* systems, AngII-induced induction of proinflammatory mediators required the presence of RAGE. Furthermore, using three different AngII-dependent mouse models of atherosclerosis, RAGE deletion attenuated the development of atherogenesis. To investigate a possible interaction between the two receptors, we then used the Receptor-HIT assay in HEK293FT cells to monitor the proximity between RAGE and the AT_1_ receptor. We found AngII-induced recruitment of β-arrestin2/Venus to RAGE/Rluc8, confirming the formation of a heteromeric complex between the two receptors. Using more traditional BRET saturation assays [45] we further confirmed the specificity of the interaction between the two receptors, and showed that it was decreased following treatment with AngII, while a BRET trafficking assay [46] revealed that RAGE underwent cellular trafficking upon AngII-induced activation of the AT_1_ receptor.

## RTK-RTK Receptor-HIT

Unlike GPCRs, most of which are believed to be functional as monomers, most RTKs require oligomerisation for activation and signalling [4], including homodimerisation and heterodimerisation, with increasing evidence of higher-order oligomer formation as well [47]. The ErbB/HER family of RTKs are one of the most well studied signalling families, and comprise four members: (i) EGFR/erbB1/HER1, (ii) erbB2/HER2/NEU, (iii) erbB3/HER3, and (iv) erbB4/HER4, which are known to undergo both homo- and hetero-dimerisation with one another [48]. Of the four, HER3 is unique in that it is kinase impaired and it is now known that it requires heteromerisation for transduction of signalling [48,49]. We used the Receptor-HIT assay in HEK293FT cells to investigate the heteromer that forms between the EGFR and HER3 [50]. As expected for the kinase-inactive HER3, we found negligible recruitment of Grb2/Venus to HER3/Rluc8 when treated with its ligand heregulin (HRG) or with EGF. However, when it was coexpressed with the unlabelled EGFR in the Receptor-HIT assay, there was robust HRG and EGF dose-dependent recruitment of Grb2/Venus to HER3/Rluc8. This confirmed the close proximity of the two receptors and revealed that heteromerisation with EGFR is required for recruitment of the Grb2 signalling protein to HER3. In the reverse configuration, EGF was able to robustly recruit Grb2/Venus to EGFR/Rluc8, however coexpression of unlabelled HER3 was required for HRG-induced recruitment of Grb2/Venus. Inhibition of EGFR with AG 1478 inhibited all of the above Grb2/Venus recruitment signals, further confirming that heteromerisation with active and functional EGFR is required for transduction of HER3 signalling. Overall, this study confirmed the existence of the EGFR-HER3 heteromer and highlighted the adaptability of the Receptor-HIT assay beyond GPCR heteromers.

## Extracellular Receptor-HIT

**Figure 2.**
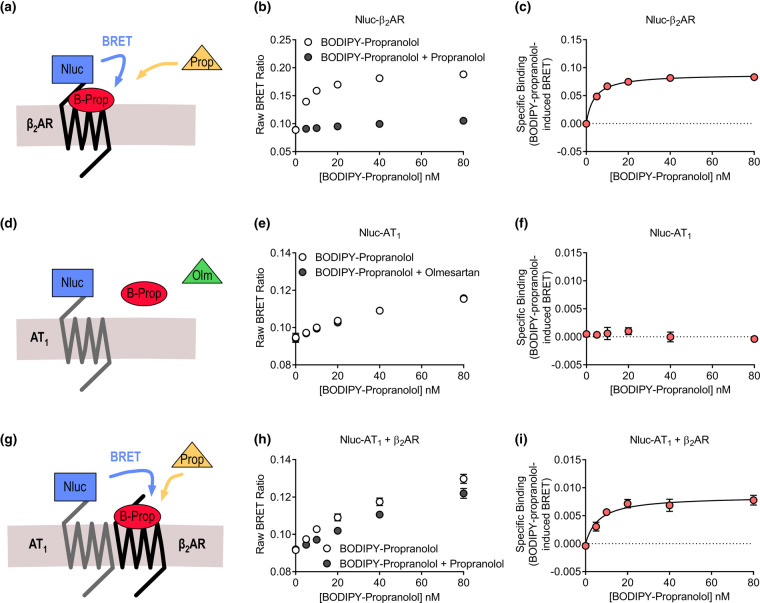
Extracellular Receptor-HIT assay. HEK293FT cells expressing Nluc/β_2_AR (**a**–**c**) or Nluc/AT_1_ (**d**–**f**) or Nluc/AT_1_ + β_2_AR (**g**–**i**) were treated with BODIPY-propranolol (B-Prop) in the presence or absence of 10 µM propranolol (Prop; **a**–**c**, **g**–**i**) or the presence or absence of 1 µM olmesartan (Olm; **d**–**f**) to generate BODIPY-propranolol total and non-specific binding data (**b**, **e**, **h**) and specific binding curves (where possible; **c**, **f**, **i**). Data are presented as the raw BRET ratio (**b**, **e**, **h**) or specific binding (**c**, **f**, **i**), mean ± SEM of three (**e**, **f**), four (**b**, **c**) or eight (**h**, **i**) independent experiments. Figure reproduced from [53].

Receptor-HIT has most commonly been used to investigate interactions between receptors at their intracellular domains, using cytosolic proteins as the labelled interacting biomolecule. However, it is also possible to use Receptor-HIT to investigate interactions that occur at extracellular surfaces of receptors. In this form of the assay, the interacting biomolecule is a ligand labelled with the complementary reporter component [51]. To do this, we adapted our NanoBRET ligand binding assay [52], which enables monitoring and quantification of binding of fluorescently labelled ligands to Nanoluciferase-(Nluc)-labelled receptors, in live cells and in real time. We recently published this Receptor-HIT ligand binding assay using two established heteromers [53], the heteromer between the AT_1_ receptor and the β_2_ adrenergic receptor (β_2_AR) [20,54,55], and between the AT_1_ receptor and angiotensin II type 2 (AT_2_) receptor [18,56–58]. For the AT_1_-β_2_AR heteromer [53], we first monitored binding of the BODIPY-630/650 tagged antagonist propranolol (BODIPY-propranolol) to Nluc/β_2_AR (Figure 2a) and observed saturable binding that was displaced by unlabelled propranolol (Figure 2b), enabling generation of a specific binding curve (Figure 2c). We tested binding of BODIPY-propranolol to Nluc/AT_1_ (Figure 2d), but did not observe any specific binding that could be displaced by the AT_1_ antagonist olmesartan (Figure 2e,f). Next, using the Receptor-HIT assay, we coexpressed unlabelled β_2_AR with Nluc/AT_1_ (Figure 2g), and again observed saturable binding that could be displaced by unlabelled propranolol (Figure 2h), enabling generation of a specific binding curve (Figure 2i). This Receptor-HIT signal confirmed the close proximity of the two receptors at their N terminal domains, indicating their heteromerisation. We also confirmed that the Receptor-HIT signal was not simply due to bystander BRET as a result of overcrowding of receptors at the cell surface, as we did not observe a similar signal with Nluc/CCK_1_, which was expressed at similar levels to Nluc/AT_1_. We also conducted the Receptor-HIT assay in the reverse configuration, coexpressing unlabelled AT_1_ receptors with Nluc/β_2_AR and treating with BODIPY-630/650 tagged AngII (BODIPY-AngII). In these cells, we observed saturable binding of BODIPY-AngII that could be displaced with olmesartan, enabling generation of a specific binding curve. Interestingly, in both Receptor-HIT assays, we saw a small but significant decrease in the affinity of binding to AT_1_-β_2_AR heteromers when compared with binding to the individual monomeric/homomeric receptor, suggesting that the unbound receptor may be negatively allosterically modulating the bound receptor, resulting in reduced fluorescent ligand binding affinity.

We also used the Receptor-HIT ligand binding assay to investigate the AT_1_-AT_2_ heteromer [53]. In these assays, we used TAMRA-labelled AngII (TAMRA-AngII), and because this ligand binds to both receptors within the heteromer, a more complex assay set up was required. Here, the Nluc-labelled receptor was blocked from binding TAMRA-AngII with the use of a selective antagonist. This enabled monitoring of TAMRA-AngII binding specifically to the unlabelled receptor, and we confirmed this by competitive binding of a selective ligand for the unlabelled receptor. This result highlights the broad applicability of the Receptor-HIT ligand binding assay, even in situations where non-selective labelled ligands are used.

The Receptor-HIT ligand binding assay has also been used with time-resolved FRET (TR-FRET) instead of BRET [59]. Hounsou et al. [59] investigated ligand binding to the dopamine D_1_-D_3_ heteromer by coexpressing SNAP-tag-labelled D_1_ receptors with unlabelled D_3_ receptors. They monitored binding of a fluorescent D_3_ receptor-selective ligand, and used unlabelled D_3_ selective ligands to displace that binding and disrupt the FRET signal. One limitation of the TR-FRET approach in comparison with the BRET approach, is that the TR-FRET assay requires wash steps to remove the SNAP-tag substrate. In contrast, the BRET assay can be performed entirely homogenously, reducing time and potential introduction of error.

## Receptor-HIT with endogenous proteins

Receptor-HIT requires that two components within the system be labelled (one receptor and one interacting biomolecule). As at least one of the these (the receptor) is a protein (and two in the case of the intracellular Receptor-HIT assay), this means that traditionally, Receptor-HIT assays have required the exogenous expression of transfected proteins genetically fused to appropriate labels (fusion proteins). However, with the advent of the clustered regularly interspaced short palindromic repeats (CRISPR/Cas9) tool for modifying genomic loci, the ability to genetically fuse labels to endogenous proteins has become much more feasible [60,61]. Using endogenous proteins that have been labelled with CRISPR/Cas9-mediated homology-directed repair has many advantages over more conventional transient or stable transfection of fusion proteins. Unlike exogenously expressed fusion proteins, expression of endogenous fusion proteins is controlled by normal regulators of transcription and translation, and their expression is driven by endogenous promotion. Furthermore, transient expression systems result in heterologous populations of cells with varying levels of fusion proteins, unlike stable or endogenous expression systems that consist of homogenous cell populations.

**Figure 3.**
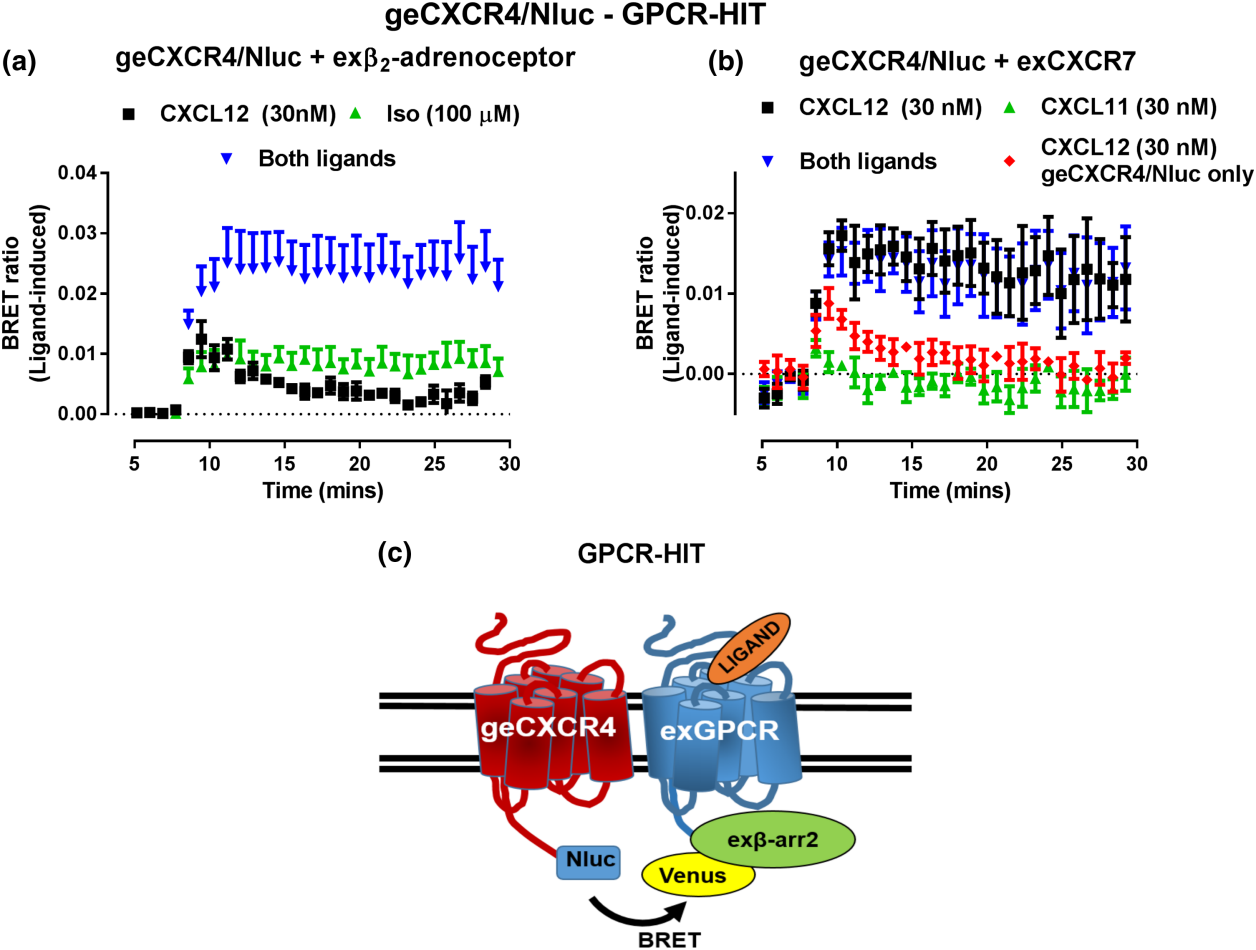
Receptor-HIT assay using an endogenous CRISPR/Cas9-engineered receptor. CRISPR/Cas9 genome-editing was used to genetically fuse Nluc to the C terminus of CXCR4 (geCXCR4/Nluc) in HEK293FT cells. (**a**) Coexpression of exogenous unlabelled β_2_AR (exβ_2_-adrenoceptor) and β-arrestin2/Venus resulted in isoprenaline-(Iso)-induced recruitment of β-arrestin2/Venus proximal to geCXCR4/Nluc. (**b**) CXCL12 (which binds both CXCR4 and CXCR7) treatment caused transient β-arrestin2/Venus recruitment proximal to geCXCR4/Nluc (red), however this became sustained upon coexpression of exogenous unlabelled CXCR7 (exCXCR7; black). CXCL11 (which is CXCR7 specific) induced weak and very transient recruitment of β-arrestin2/Venus. (**c**) Illustration of the Receptor-HIT assay using geCXCR4/Nluc, unlabelled exogenous receptor (exGPCR) and β-arrestin2/Venus. Data are presented as the ligand-induced BRET ratio (calculated by subtracting vehicle treated samples from ligand treated samples), mean ± SEM of three independent experiments. Ligand added at ∼8 min. Figure reproduced from [17].

In the first-ever study using BRET to monitor the pharmacology of endogenous proteins, we generated HEK293FT cells expressing the genome-edited CXCR4 chemokine receptor C-terminally tagged with Nluc [17]. We used these cells to assess various aspects of CXCR4 pharmacology, including Receptor-HIT to investigate the previously published heteromer with β_2_AR [62]. We observed that activation of unlabelled β_2_AR with isoprenaline resulted in recruitment of β-arrestin2/Venus proximal to the genome-edited CXCR4/Nluc, confirming the close proximity of the two receptors (Figure 3a). As a control, the unlabelled vasopressin 1b (V_1b_) receptor was also coexpressed in the genome-edited CXCR4/Nluc cells in the Receptor-HIT configuration, but no Receptor-HIT signal was generated upon vasopressin-induced activation, despite both the β_2_AR and the V_1b_ receptor being expressed at similar levels (as gauged by agonist-induced β-arrestin2 recruitment to each receptor). The genome-edited CXCR4/Nluc cells were also used to investigate the previously published heteromer with CXCR7 [63,64]. We found that CXCL12 induced transient β-arrestin2/Venus recruitment to genome-edited CXCR4/Nluc, but when unlabelled CXCR7 was coexpressed, and noting CXCL12 activates both CXCR4 and CXCR7, β-arrestin2/Venus underwent sustained recruitment (Figure 3b). This study demonstrated that the Receptor-HIT assay can be used to investigate heteromers that include endogenous receptors, despite them being expressed at low, physiological expression levels.

## Conclusion

The Receptor-HIT assay enables detection and characterisation of receptor heteromers in live cells and in real time. It can be used to investigate heteromers composed of all classes of receptors, and can monitor receptor interactions that occur both at intracellular and extracellular domains. Receptor-HIT can also be used to detect heteromers that include genome-edited endogenous receptors, expressed at low physiological levels. Drug discovery assays such as Receptor-HIT can produce early-stage discoveries that can eventually be translated to clinical outcomes, as demonstrated with the AT_1_-CCR2 heteromer.

## Perspectives

- Receptor heteromers are macromolecular complexes that can have novel pharmacological properties compared with their constituent receptors, expanding the complexity of receptor signalling systems.
- The Receptor-HIT assay is an early-stage drug discovery assay that enables the detection and characterisation of receptor heteromers.
- Future research with assays such as Receptor-HIT will expand the number of receptor heteromers that can potentially be therapeutically targeted, with the aim of providing greater specificity and selectivity compared with targeting the individual constituent receptors.

## Open Access Statement

Open access for this article was enabled by the participation of University of Western Australia in an all-inclusive Read & Publish pilot with Portland Press and the Biochemical Society under a transformative agreement with CAUL.

## Abbreviations

AT_1_: angiotensin II (AngII) type 1
BRET: bioluminescence resonance energy transfer
EGFR: epidermal growth factor receptor
FSGS: Focal Segmental Glomerulosclerosis
GPCRs: G protein-coupled receptors
HRG: heregulin
RAGE: receptor for advanced glycation end-products
RTKs: receptor tyrosine kinases
STNx: subtotal-nephrectomised
